# Noncoding RNA Regulation of Dopamine Signaling in Diseases of the Central Nervous System

**DOI:** 10.3389/fmolb.2016.00069

**Published:** 2016-10-25

**Authors:** William T. Carrick, Brandi Burks, Murray J. Cairns, Jannet Kocerha

**Affiliations:** ^1^Department of Chemistry, Georgia Southern UniversityStatesboro, GA, USA; ^2^School of Biomedical Sciences and Pharmacy and the Hunter Medical Research Institute, University of NewcastleCallaghan, NSW, Australia; ^3^Schizophrenia Research InstituteSydney, NSW, Australia

**Keywords:** microRNAs, psychiatric, neurodegeneration, noncoding RNA, dopaminergic, schizophrenia, dopamine, epigenetic

## Abstract

Dopaminergic neurotransmission mediates a majority of the vital central nervous system functions. Disruption of these synaptic events provokes a multitude of neurological pathologies, including Parkinson's, schizophrenia, depression, and addiction. Growing evidence supports a key role for noncoding RNA (ncRNA) regulation in the synapse. This review will discuss the role of both short and long ncRNAs in dopamine signaling, including bioinformatic examination of the pathways they target. Specifically, we focus on the contribution of ncRNAs to dopaminergic dysfunction in neurodegenerative as well as psychiatric disease.

## Introduction

Dopamine is a potent neurotransmitter in the central nervous system that governs a diverse panel of neuronal functions. Accumulating studies indicate that noncoding RNAs (ncRNAs) modulate almost all aspects of dopamine signaling, including its receptors, and transporter, as well as differentiation and viability of dopaminergic neurons. The processing and transcription of those implicated ncRNAs are also subject to control. Moreover, there are emerging roles for both short and long ncRNAs (lncRNAs) in synaptic transmission, including miRNAs, and antisense transcripts. This review discusses ncRNA-mediated mechanisms which influence dopaminergic signaling under control and pathogenic conditions.

The diverse classes of ncRNAs target all stages in the lineage from stem cells to mature dopaminoceptive neurons (DN) which express dopamine receptors. One of the first ncRNAs reported to modulate midbrain DN (mDN) differentiation and viability was miR-133b (Kim et al., 2007). A subset of additional miRNAs are also now known to direct DN fate, including miR-132, miR-135a2, and miR-218 (Yang et al., 2012; Anderegg et al., 2013; Baek et al., 2014). Moreover, studies indicate that selected miRNAs, such as miR-132, influence DN maturation, and survival by binding to key neuronal transcription factors, including Nurr1 (Yang et al., 2012). Reports also suggest that miRNA processor genes, such as Dicer, are required for terminal differentiation of embryonic stem cells (ESC) into DN (Kim et al., 2007). Dicer cleaves the mature miRNA from its precursor transcript. Mouse studies revealed that Dicer depletion in dopaminoceptive neurons alters brain morphology and neurobehavioral patterns (Cuellar et al., 2008). Further, ablation of Dicer in mouse mDN led to nearly 90% cell death by 8 weeks of age; however, that level of degeneration was not seen when Dicer was depleted in neurons receiving input from dopamine-transporter expressing cells (Kim et al., 2007). These data suggest that miRNA processing deficits may impact specific classes of mature neurons more potently.

It is now evident that ncRNAs target expression of the receptors which bind dopamine. There are at least 5 reported subtypes of dopamine receptors (D1R-D5R), however, D1R, and D2R are the most abundant (Greengard, 2001; Girault and Greengard, 2004). Protein levels of D1R and D2R are well known to fluctuate during the normal aging process as well as in numerous neuronal pathologies (Hurley et al., 2001, 2003; Kaasinen and Rinne, 2002; Rangel-Barajas et al., 2015). *In vitro* analysis of varying deletion constructs for the D1R gene identified regions of putative miRNA binding sites within the D1R 3′UTR. It was discovered that a short 94 bp segment of the 3′UTR was adequate for post-transcriptional regulation and contains a miR-142-3p binding site (Tobón et al., 2012). Correspondingly, similar studies have also investigated ncRNA control of D2R. Antisense-knockdown of neuronally-enriched miR-9 and miR-326 provoked upregulated expression of D2R 3′UTR in luciferase based assays (Shi et al., 2014). It is important to note, however, that D1R and D2R pathways are also intricately connected and their receptors can even form heteromers (Hasbi et al., 2009; Perreault et al., 2011a,b, 2014a,b). Thus, a subset of ncRNAs may influence signaling of multiple receptor types.

It is well established that dopamine receptor signaling is synchronized with excitatory and inhibitory responses triggered by other neurotransmitters. The role of ncRNAs in mediating this cross-talk, however, is undefined. Saba et al suggests miR-181a as a candidate with a pleiotropic role in neurotransmission, as its expression is modulated by dopamine while it also targets the glutamate receptor subunit GluA2 (Saba et al., 2012). Notably, glutamatergic transmission emerges through AMPA, Kainate, NMDA, and metabotropic receptor classes, underscoring the complexity of synaptic physiology (Planells-Cases et al., 2006). Orchestration of this extensive neurotransmission network by ncRNAs may reveal enduring mechanistic questions that have stymied drug development for many neurological pathologies.

Dopamine signaling is ubiquitous throughout the brain, however, this pathway also functions in select tissues outside of the central nervous system, including the cardiovascular system and kidney (Lokhandwala and Barrett, 1982; Lokhandwala and Amenta, 1991). Impaired dopamine-signaling in the kidney is a primary contributor to hypertension (Banday and Lokhandwala, 2008; Chugh et al., 2012, 2013). Correspondingly, hypertension is repeatedly linked to neuronal dysfunction, including cognitive deficits in patients with Alzheimer's Disease. Investigating ncRNA regulation of dopamine-mediated hypertension in the kidney could reveal novel origins of neurological disease. A recent study identified five dysregulated miRNAs in renal cells from patients carrying hypertension-associated polymorphisms (SNP) in the D2R gene (Han et al., 2015). Of those 5 misregulated miRNAs in the SNP-DR2 cells, miR-217 expression was directly connected to D2R expression through a TGF β1-Wnt5a-Ror2 feedback loop. In a separate study, Zhang et found that let-7d silenced dopamine-receptor 3 (D3R) in renal cells at the chromatin level through histone-3 –lysine-9 dimethylation (Zhang et al., 2016). However, these studies did not explore whether specific disruption of these miRNAs induce hypertension and associated neuronal damage.

**Figure 1:**
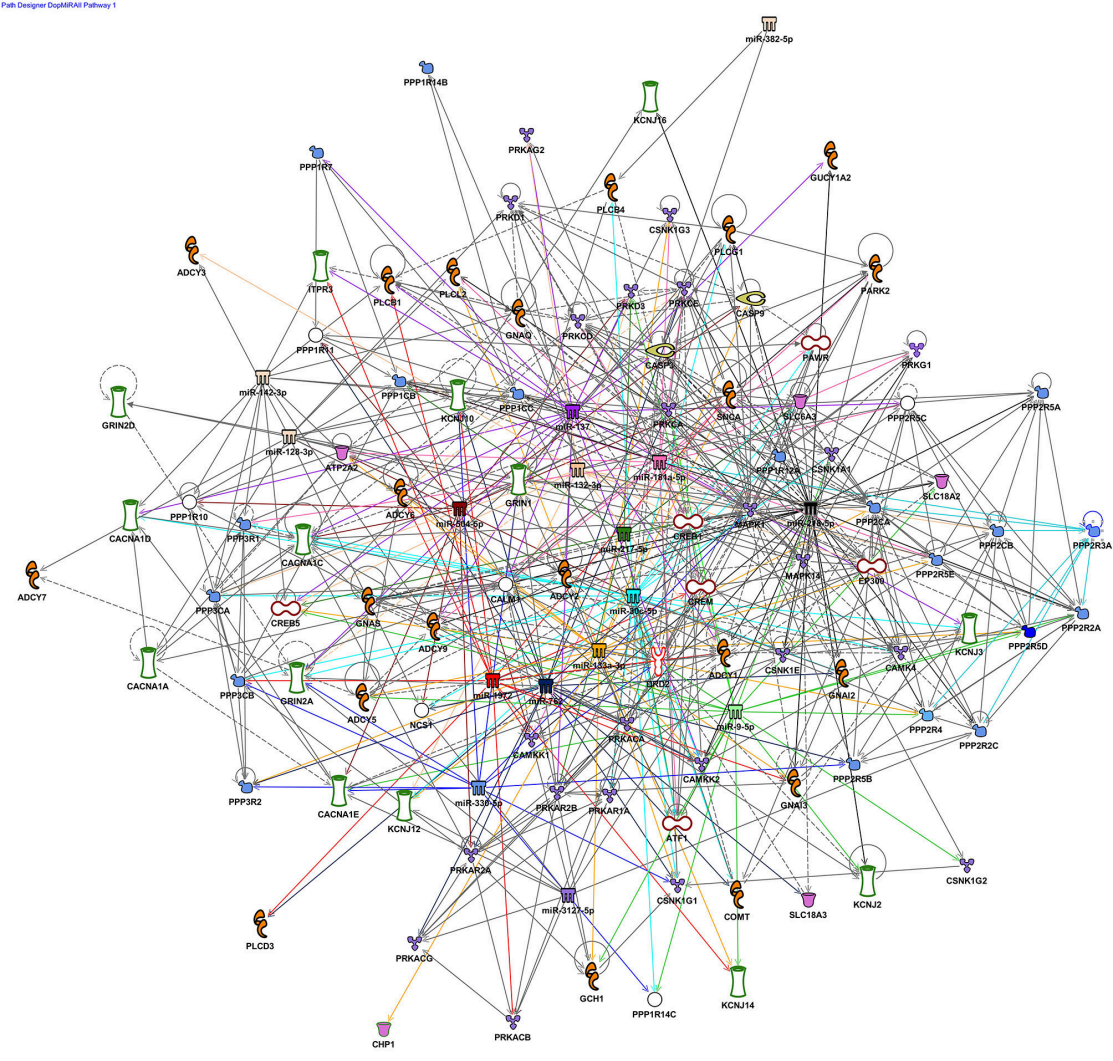
**Dopaminergic miRNA nodes in a rich network of connections in synaptic plasticity pathways**. Ingenuity pathway analysis (IPA) was used to explore the molecular connectivity predicted between miRNA implicated in dopaminergic development, function and disease (references for all implicated miRNAs examined in this figure are listed in Table 1). miRNA targets, both experimentally validated and predicted by TargetScan were filtered with respect to dopamine-associated canonical pathways and used to generate a network graph. Core miRNA nodes near the center of the graph are color-coded and have colored edges to denote their connections. The largest network defined by the filter was *Dopamine-DARPP32 in cAMP Signaling* (85 genes) followed by *Dopamine Receptor Signaling (40)*. These networks were also enriched with synaptic plasticity pathways, *Synaptic LTP/LTD* (43/29 genes), CREB Signaling in Neurons (43 genes), and the *Axon Guidance Signaling (27 genes)*.

This review focuses on neuronal ncRNA mediators of dopamine signaling, although, it is evident this epigenetic pathway also exerts significant influence in other cell types. We outline a subset of these ncRNAs which are implicated in neurodegenerative and neuropsychiatric pathologies Table 1). Additionally, we bioinformatically examined the pathways targeted by the reported miRNAs. Using Ingenuity Pathway Analysis (IPA), several key dopamine-associated pathways were identified, including *Dopamine-DARPP32 in cAMP Signaling, Dopamine Receptor Signaling, Synaptic LTP/LTD, CREB Signaling in Neurons*, and *Axon Guidance Signaling* (Figure 1). Overall, the published findings suggest the entire neurotransmission cascade activated by dopamine, as well as its misregulation, is guided by ncRNAs.

**Table 1:** **Dopamine-responsive miRNAs and their association with neurological functions/disease**.

**Component of Dopamine pathway targeted by miRNA**	**miRNA (or miRNA processor) implicated**	**Disease (or function) miRNA is associated with**	**References**
Dopaminergic neuronal development	miRs–132, 133b, 135a2, 218; Dicer	differentiation and viability of dopaminergic neurons	Kim et al., 2007
D1R (dopamine-1-receptor)	miR-142-3p	binds D1R 3′UTR	Tobón et al., 2012
	miR-128	Parkinson's Disease (with ERK2 regulatory loop)	Tan et al., 2013
	miR-382	alcohol addiction	Li J. et al., 2013
	miR-504	(1) polymorphism in miRNA binding site linked to nicotine addiction (2) depression	(1) Huang and Li, 2009 (2) Zhang et al., 2013
D2R (dopamine-2-receptor)	miR-9 and miR-326	(1) Binds D2R 3′UTR (2) depression	(1) Shi et al., 2014 (2) Zhang et al., 2015
	miR-137	Parkinson's Disease	Kong et al., 2015
	miR-217	Regulates D2R in kidney; polymorphism in miRNA binding site linked to hypertension	Han et al., 2015
	Ago2 (Argonaute 2)	Cocaine addiction	Schaefer et al., 2010
	Dgcr8	22q11 deletion syndrome/schizophrenia	Chun et al., 2014
D3R	Let-7d	Binds D3R 3′UTR (kidney)	Zhang et al., 2016
DAT (dopamine transporter)	miRs-30b-5p, 1301, 1972, 6070	miRNA binding sites located in polymorphic region associated with Attention Deficit Hyperactivity Disorder	Sery et al., 2015
	miRs-762, 1266, 3127, 3192, 4259	miRNA binding sites located in polymorphic region associated with Bipolar Disorder	Pinsonneault et al., 2011
Neurotransmitter signaling crosstalk	miR-181a	Crosstalk between dopamine and AMPA signaling	Saba et al., 2012
Dopaminergic neuronal markers (i.e. Nurr1, TH, Pitx3)	Ago2	Morphine administration - dysregulates a regulatory loop of; Ago2, Nurr1, Pitx3 and TH, and miR-133b	Sanchez-Simon et al., 2010
	miR-133b	Parkinson's Disease -regulatory loop with miR-133b and Pitx3	Kim et al., 2007
		schizophrenia- regulatory loop with miR-133b, Nurr1, Pitx3, TH, and D1R	Song et al., 2012
		cocaine addiction –regulatory with miR-133b, Pitx3, TH, and D1R/D2R	Barreto-Valer et al., 2012

## miRNA-mediated regulation of dopamine signaling in neurological disease

### Dopamine receptors and miRNAs in neurological disease

All five classes of dopamine receptors are putative miRNA targets in neurological pathologies, although D1R and D2R are the most interrogated to date. In neurodegenerative Parkinson's Disease (PD), these patients classically display upregulated expression of D1R and D2R with hypersensitive response to dopamine (Seeman and Niznik, 1990; Kaasinen et al., 2000; Hurley et al., 2001; Gerfen et al., 2002; Zhu et al., 2002; Chu et al., 2004; Hisahara and Shimohama, 2011). Studies by Tan et al revealed that genetic knockout of miR-128 in D1-neurons of wild-type (WT) mice enhances D1R-activation by a dopamine agonist and provokes increased ERK2 expression (Tan et al., 2013). Parallel responses of D1R-hyperexcitablity/ERK2 in miR-128 deficient mice and PD patients suggests this miRNA is an active mechanism underlying PD pathology. In a separate study, a drosophila model which recapitulates PD through common genetic variants of alpha-synuclein uncovered a dysfunctional D2R-miR-137 pathway (Kong et al., 2015). Expression of miR-137 was elevated in the PD drosophila and anti-correlated with D2R protein levels, consistent with miRNA target regulation. Subsequently, miR-137 binding sites in the D2R gene were validated through *in vitro* analyses.

Targeting of dopamine receptor signaling by physiological stressors that trigger depression and related neuropsychiatric phenotypes is guided by select miRNAs (Zhang et al., 2013, 2015). Early life stress (ELS) can broadly impact normal development of the dopamine system, leading to inadequate coping methods for chronic-unpredictable stress (CUS) and depression in adults. Expression profiles of D1R, miR-504 (a D1R target), and D2R were explored in rats with ELS modeled through maternal deprivation (MD) coupled with exposure to CUS at mature age (MD/CUS) (Zhang et al., 2013). The MD/CUS rats indeed showed significant correlation between D1R, D2R, miR-504 as well as correlation of D2R and miR-504 with depression-related behaviors. In a separate study also conducted by Zhang et al. they subsequently identified several miRNAs (miR-9, miR-200a, miR-141, and miR-326) that target the 3′-UTR of the D2R (Zhang et al., 2015). *In vitro* analyses confirmed that miR-9 binds the D2R transcript (Zhang et al., 2015). They also found that miR-9, miR-326, and D2R correlated with depressive-like phenotypes in mature MD/CUS rats. In addition, aberrant expression of miR-326 in the MD/CUS rats was normalized with the anti-depressant escitalopram (Zhang et al., 2015). These results not only implicate dopamine-regulated miRNAs in depression, but also suggests these ncRNAs may mediate treatment response to anti-depressants.

Targeting of dopamine receptors by miRNAs was also uncovered in a model for addiction (Li J. et al., 2013). Post-administration of alcohol to rats, the NAc in the basal forebrain displayed repressed levels of miR-382 levels while its bioinformatic target, D1R, was upregulated. Cell-based studies as well as site-specific knockdown and overexpression of miR-382 in rat NAc confirmed it targets the D1R gene (Li J. et al., 2013). Moreover, miR-382 overexpression altered the neuronal excitability responses of D1R and diminished desire to consume alcohol. This report makes the direct connection between miRNA regulation of D1R and neuropsychiatric phenotypes in an animal model.

Disease-associated polymorphic regions in the dopamine receptor genes can impact miRNA binding to their target sites. One such reported example is the D1R rs686 polymorphism linked to nicotine dependence (Huang and Li, 2009). Two miRNA sites were bioinformatically identified within the rs686 region, miR-504 and miR-296. Further, miR-504 regulation of D1R was confirmed *in vitro*. Notably, miR-504 appears to exhibit stronger binding potential to the rs686 allele compared to the wild-type (WT) allele, which could alter the allelic representation of DR1.

### Dopamine transporter (DAT) and miRNAs in neurological disease

The dopamine transporter gene (DAT/SLC6A3) is critical for removal of dopamine in the synapse to halt neurotransmission (German et al., 2015; McHugh and Buckley, 2015). Correspondingly, the disruption of this gene reportedly provokes numerous distinct neuronal pathologies (German et al., 2015; McHugh and Buckley, 2015). Two separate studies investigated miRNA binding in polymorphic regions of the DAT gene. One study examined a 40-base pair 3′UTR region within the DAT transcript linked to Attention Deficit Hyperactivity Disorder (ADHD) (Sery et al., 2015). Bioinformatic analysis of this 40-base pair segment identified putative sites for miRs-1972, 30b-5p, 1301, and 6070 (Sery et al., 2015). A second study focused on a DAT polymorphism linked to bipolar disease, rs27072 (Pinsonneault et al., 2011). The rs27072 analysis revealed binding sequences for miRs-762, 4259, 3192, 3127, and 1266 (Pinsonneault et al., 2011). Collectively, although these findings are only predictive at this point, they provide a framework for future investigation.

In addition to dopamine, DAT can also transport the neurotoxin 6-hydroxydopamine (6-OHDA), eliciting phenotypes which recapitulate PD (Westerlund et al., 2010). 6-OHDA kills dopaminergic neurons, providing a platform to identify and study dopamine-regulated ncRNAs. A subset of miRNAs are reported to mediate 6-OHDA neuronal damage, including miRs-124, 126, 668-3p, let-7d-3p, 3077-3p, 665-5p, 99b-3p, 323-3p, 875, 207, 425-5p, 19b-3p, and 338-3p (Li L. et al., 2013; Kim et al., 2014; Saraiva et al., 2016).

### Dopamine signaling and miRNA processing machinery in neurological disease

The processing of miRNAs involves several stages, and their disruption is pathogenic (Kocerha et al., 2009, 2015; Chan and Kocerha, 2012; Chun et al., 2014). The primary-miRNA is cleaved by Dgcr8 and Drosha, followed by second cleavage with Dicer into the mature transcript (Bartel, 2004, 2009; Macias et al., 2013). The mature miRNA gets assembled, along with argonaute proteins, into RISC for target gene regulation (Bartel, 2004, 2009). Studies by Schaefer et al found that deficiency of the RISC-associated Argonaute 2 (Ago2) protein in D2R-neurons significantly reduced self-administration of cocaine in mice (Schaefer et al., 2010). This Ago2 mouse model was then used to compile a list of D2R-localized miRNAs which putatively control cocaine addiction.

Ago2 expression is also reportedly regulated by the pain medication morphine (García-Pérez et al., 2013). Acute administration of morphine in rats led to increased Ago2 levels as well as the disruption of a panel of dopaminergic markers such as Nurr1, Pitx3, and TH activity. Pitx3 and TH are mentioned below as part of a conserved miRNA mechanism with miR-133b in neurological disease. Notably, pain relief is mediated by dopamine release in the midbrain, a region where miR-133b functions are reported (Kim et al., 2007; Navratilova et al., 2015). Indeed, a separate study revealed morphine alters miR133b-Pitx3 expression and differentiation of dopaminergic neurons in a zebrafish model (Sanchez-Simon et al., 2010). These results suggest ncRNAs which are regulated by morphine influence neurotransmission.

The gene for the core miRNA processor, Dgcr8, is located within the 22ql1 chromosomal locus, a region persistently associated with schizophrenia and other neurological anomalies (Merico et al., 2015; Zhao et al., 2015). Studies by Chun et al showed that a mouse model for the 22q11 deletion impedes Dgcr8 function, leading to enhanced expression of D2R, and disruption of corticothalamic synaptic transmission (Chun et al., 2014). Moreover, the aberrant D2R signaling provokes a hypersensitive response to antipsychotics used for schizophrenia treatment. Identifying the specific miRNAs impacted by haploinsufficient Dgcr8 processing in the 22q11 mice would facilitate future studies. Importantly, these data also suggest ncRNA mechanisms can influence drug efficacy and medical outcomes.

### Conserved dopamine-mediated miRNA mechanisms in disease; the miR-133b-Pitx3 example

A shared characteristic of many neurological diagnoses is impaired synaptic transmission; thus, it is pertinent to uncover conserved dopamine-linked ncRNA mechanisms in brain pathologies (Henstridge et al., 2016). One conserved ncRNA pathway was identified through miRNA expression profiles of midbrain dopamine neurons (mDNs) in PD patients (Kim et al., 2007; Briggs et al., 2015). One of the dysregulated miRNAs, miR-133b, controls mDN differentiation through a feedback loop with Pitx3 (Kim et al., 2007). Pitx3 is a transcription factor essential for DN survival (Bergman et al., 2010). A separate study, however, reported that miR-133b expression is not impacted within the substantia nigra region of the midbrain (Schlaudraff et al., 2014). These results suggest the presence of site-specific miRNA mechanisms, providing an additional layer of dopaminergic influence.

The Pitx3-miR-133b pathway associated with PD is also implicated in the neuropsychiatric disorder schizophrenia. Song et al reported that Pitx3-miR-133b is dysregulated in mice with schizophrenia phenotypes provoked through overexpression of the stress protein heme-oxygenase-1 (HO-1) (Song et al., 2012). Moreover, these mice exhibit disrupted profiles of dopaminergic markers D1R, Nurr1 and tyrosine hydroxylase (TH), as well as neurotransmitters serotonin and dopamine. Parallel changes of dopamine and serotonin in this model is consistent with extensive cross-talk reported in synaptic signaling (Henstridge et al., 2016). Further, an independent study found this miRNA pathway is altered in another neuropsychiatric disorder, cocaine addiction (Barreto-Valer et al., 2012). Zebrafish embryos were exposed to cocaine followed by gene expression analysis at various developmental timepoints. In addition to dysregulated levels of miR-133b, Pitx3, D1R, TH in these embryos, there were also significant changes in D2R (Barreto-Valer et al., 2012). Collectively, these data suggest subsets of epigenetic mechanisms are conserved across neurological diseases and, further, may contribute to a general trend of synaptic dysfunction in these pathologies.

## lncRNA regulation of dopamine signaling in the brain

Although miRNAs are the most studied to date, the detection of neuronal lncRNAs is expanding. One of the first annotated lncRNAs in the central nervous system, BC1, mediates D2R expression *in vivo* and related electrophysiological responses (Wang et al., 2002; Centonze et al., 2007; Zhong et al., 2009; Maccarrone et al., 2010). BC1 knockout mice displayed significant upregulation of D2R protein in the striatum and potentiated responses to D2R agonists (Centonze et al., 2007). Moreover, although BC1 was reported to act as a translational repressor, that is not the mechanism in which it regulates D2R (Wang et al., 2002; Centonze et al., 2007). This study suggests BC1 may have additional functions that have yet to be investigated.

Recently, Carrieri et al showed that a lncRNA antisense to the Uchl1 gene (AS-Uchl1) is significantly repressed in dopaminergic neurons of PD models (Carrieri et al., 2015). The AS-Uchl1 gene is regulated by Nurr1, a core transcription factor involved in the maturation and viability of dopamine neurons (Carrieri et al., 2015). Furthermore, the AS-Uchl1/PARK5 locus is linked to certain populations of PD patients, suggesting an intricate regulatory loop between dopamine-responsive lncRNAs and disease susceptibility genes (Belin and Westerlund, 2008).

Neurodegeneration may also be controlled by lncRNAs encoded within viral transcripts. Kuan et al showed that p137, a ncRNA transcribed from the human cytomegaloviral β2.7 gene, is neuroprotective against OHDA-induced cell damage (Kuan et al., 2012). Compellingly, p137 prevented progression of PD phenotypes within dopaminergic neurons for both *in vitro* and *in vivo* OHDA models. In addition to the miRNAs discussed previously, these data suggest that a diverse range of ncRNA classes influence neurodegenerative pathways. It is also likely these ncRNAs may regulate each other through feedback loops.

Investigation of midbrain dopamine neurons (mDN), which are primary responders to drugs of abuse, revealed lncRNA modulators of addiction phenotypes (Bannon et al., 2015). Bannon et al identified a subset of dysregulated lncRNAs in mDN from post-mortem tissue of cocaine addicts (Bannon et al., 2015). One of those misregulated lncRNAs is an antisense transcript to the gene for tumor necrosis factor receptor-associated factor 3-interacting protein 2 (TRAF3IP2). The antisense RNA was localized to the nucleus of the neurons compared to the global cellular distribution of TRAF3IP2, suggestive of epigenetic control at the chromatin level.

A recent report now also suggests Gomafu, a schizophrenia associated lncRNA, modulates dopaminergic transmission (Ip et al., 2016). In Gomafu knockout (KO) mice, a significant increase in dopamine levels was detected in the brain after exposure to the psychostimulant methamphetamine (MAP). Notably, the increased dopamine was correlated with hyperactivity of the KO mice, as measured by open field and light-dark transition tests (Ip et al., 2016). This study directly links lncRNA regulation of dopamine signaling with neurobehavioral phenotypes, although the precise mechanism is not yet elucidated.

## Conclusion

Epigenetic regulation of dopamine signaling by ncRNAs is a vital component to neuronal homeostasis. Correspondingly, impaired dopaminergic transmission provokes a range of cognitive, motor, and behavioral phenotypes in CNS disorders. The untapped pharmacological potential of ncRNA modulators to normalize aberrant synaptic transmission provides a wide-open opportunity for drug discovery efforts. Furthermore, as the dopamine pathway is extensive and partially driven by undefined mechanisms, the pleiotropic capacity of many implicated ncRNAs may enhance their efficacy.

## Author contributions

WC, BB, MC, and JK all wrote this manuscript.

### Conflict of interest statement

The authors declare that the research was conducted in the absence of any commercial or financial relationships that could be construed as a potential conflict of interest.
